# 1,25-Dihydroxyvitamin D Inhibits LPS-Induced High-Mobility Group Box 1 (HMGB1) Secretion *via* Targeting the NF-E2-Related Factor 2–Hemeoxygenase-1–HMGB1 Pathway in Macrophages

**DOI:** 10.3389/fimmu.2017.01308

**Published:** 2017-10-18

**Authors:** Zebing Rao, Na Zhang, Ning Xu, Ying Pan, Mengjun Xiao, Junxian Wu, Hong Zhou, Shuo Yang, Yunzi Chen

**Affiliations:** ^1^Department of Immunology, Nanjing Medical University, Nanjing, China; ^2^Key Laboratory of Antibody Techniques of Ministry of Health, Nanjing Medical University, Nanjing, China; ^3^Department of Pathology, Nanjing Medical University, Nanjing, China; ^4^Medical Centre for Digestive Diseases, Second Affiliated Hospital of Nanjing Medical University, Nanjing, China

**Keywords:** sepsis, vitamin D, inflammation, high-mobility group box 1 protein, damage-associated molecular patterns

## Abstract

1,25-Dihydroxyvitamin D [1,25(OH)_2_D_3_] is recognized as a key mediator of inflammatory diseases, including sepsis. Clinical studies demonstrate that 1,25 (OH)_2_D_3_ protects patients from sepsis, but clinical treatment with 1,25(OH)_2_D_3_ is rare. In this study, we report that 1,25(OH)_2_D_3_ treatment has beneficial effects and improves the survival rate in LPS-induced mouse sepsis model by blocking the secretion of high-mobility group box 1 (HMGB1), a key late regulator of sepsis. LPS-induced HMGB1 secretion is attenuated by 1,25(OH)_2_D_3_ *via* blocking HMGB1 translocation from the nucleus to the cytoplasm in macrophages. 1,25(OH)_2_D_3_ can induce the expression of hemeoxygenase-1 (HO-1), which is essential for blocking HMBG1 nuclear translocation and its secretion. When siHO-1 or an HO-1 inhibitor are used, the effect of 1,25(OH)_2_D_3_ on inhibition of HMGB1 secretion is suppressed. Considering that HO-1 is a downstream gene of NF-E2-related factor 2 (Nrf2), we further confirm that Nrf2 activation can be activated by 1,25(OH)_2_D_3_ upon LPS exposure. Together, we provide evidence that 1,25(OH)_2_D_3_ attenuates LPS-induced HMGB1 secretion *via* the Nrf2/HO-1 pathway in macrophages.

## Introduction

Sepsis is common among critically ill patients and associated with considerable morbidity and mortality. Sepsis syndromes result from an exaggerated systemic inflammatory response characterized by a massive release of early mediators, such as TNF-α and IL-1β, and by the late mediator high-mobility group box 1 (HMGB1) (1, 2).

High-mobility group box 1 is a DNA-binding nuclear protein that is selected actively following cytokine stimulation and passively released during cell death (3, 4). It is the prototypic damage-associated molecular pattern molecule and has been implicated in several inflammatory disorders (2–5). HMGB1 is released by activated monocytes and macrophages (6). Studies using neutralizing antibodies for HMGB1 have verified that increased circulating levels of HMGB1 contribute to the late lethality of endotoxemia and sepsis (7, 8). HMGB1 antibodies inhibit endotoxin lethality in mice (9) and inhibit lung inflammation following airway LPS exposure (10). These findings suggest that HMGB1 may serve as a target to reduce mortality from sepsis and the mechanisms responsible for inducing and controlling HMGB1 release becomes significant.

High-mobility group box 1 contains two nuclear localization signals and two putative nuclear export signals, indicating that HMGB1 shuttles between the cytoplasm and nucleus through a tightly controlled mechanism (11). Hemeoxygenase-1 (HO-1) has been reported to suppress the translocation and secretion of HMBG1 (12, 13). Decreased HMGB1 expression through increased HO-1 production takes a protective role in several disease states, including arthritis and sepsis (14–16). Various regulatory elements have been identified in the promoter region of HO-1, such as AP-1 and NF-κb, but NF-E2-related factor 2 (Nrf2) is of particular importance. Nrf2 is a redox-sensitive master switch to induce HO-1 activation, which modulates its gene expression by binding to a recognition site in the inducible enhancers (E1) of HO-1 and heterodimerizing with activating transcription factor 4 to exert its effect (17). The Nrf2/HO-1 pathway plays an important role in the HMGB1 secretion (18–20).

Vitamin D is a fat-soluble vitamin primarily synthesized from 7-dehydrocholesterol in the skin by ultraviolet radiation. 1,25-dihydroxyvitamin D [1,25(OH)_2_D_3_] is its active form. Many studies have proved vitamin D deficiency is closely related with clinical outcomes such as mortality of sepsis, duration of mechanical ventilation, and length of stay (21). A number of observational studies show a negative association between low vitamin D levels and risk of sepsis (22–24). However, the mechanism of anti-inflammatory action of Vitamin D remains poorly understood.

In this study, we provide evidence that 1,25(OH)_2_D_3_ attenuates LPS-induced HMGB1 nuclear export and secretion in macrophage by Nrf2/HO-1 pathway. Upon LPS exposure, 1,25-dihydroxyvitamin D activates Nrf2 nuclear translocation and induces HO-1 expression, resulting in inhibition of HMGB1 secretion.

## Results

### 1,25(OH)_2_D_3_ Inhibits LPS-Induced HMGB1 Secretion in Macrophages

**Figure 1:**
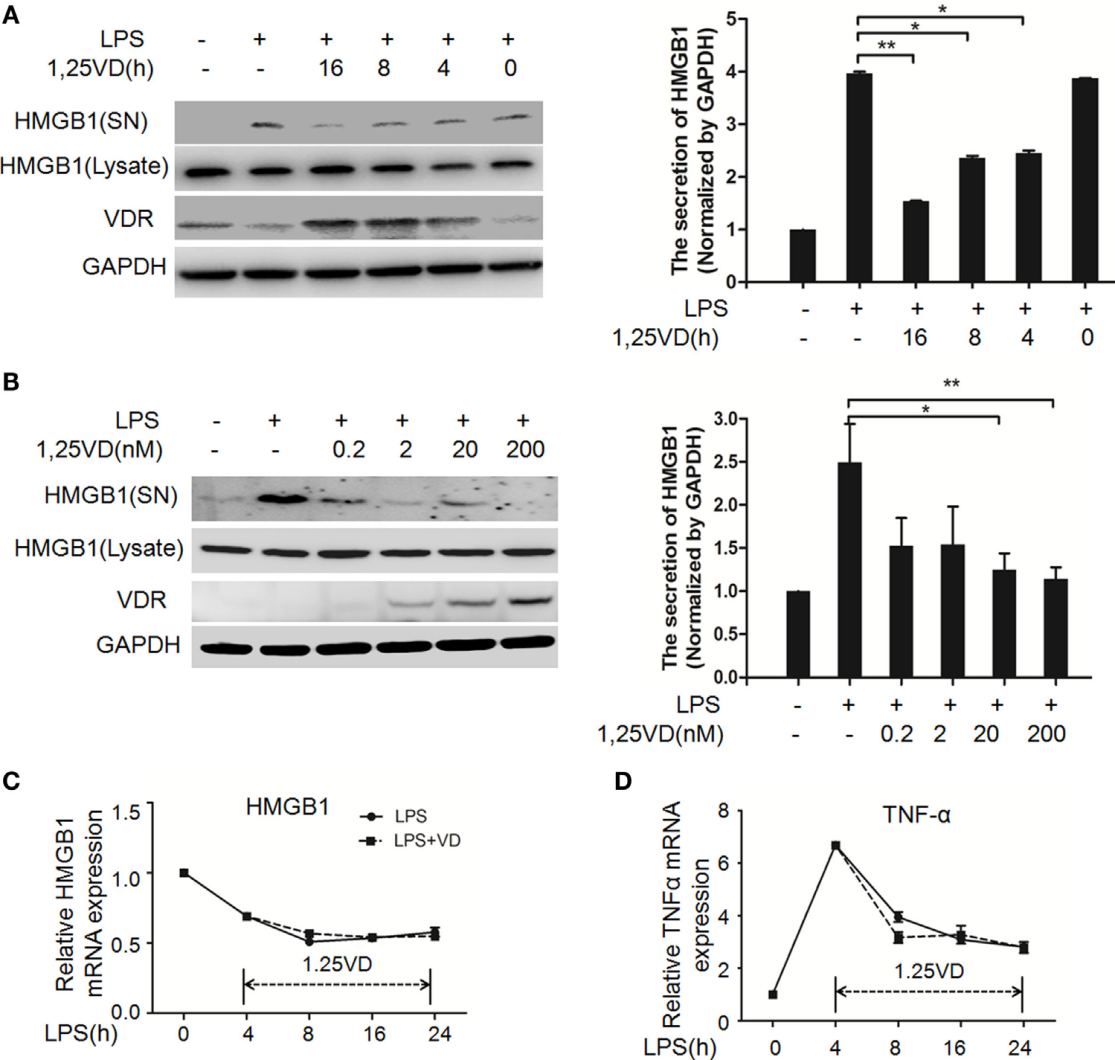
1,25-Dihydroxyvitamin D [1,25(OH)_2_D_3_] blocks LPS-induced high-mobility group box 1 (HMGB1) secretion in macrophages. **(A)** LPS-treated bone marrow-derived macrophages followed with 20 nM 1,25(OH)_2_D_3_ time course (0, 4, 8, and 16 h) stimulation or **(B)** various dose (0.2, 2, 20, and 200 nM). Supernatants (SN) and total cell extracts (Lysate) were performed by Western blotting with antibodies as indicated. Band intensity was quantified by Image J software. Data were represented as mean ± SD. *p*-Values were calculated by Student’s *t*-test: *p < 0.05, **p < 0.01, ***p < 0.001 versus LPS alone. The experiment was repeated at least three times. **(C,D)** LPS pretreatment for 4 h followed by 20 nM 1,25(OH)_2_D_3_ time course (0, 4, 8, 16, and 24 h) stimulation. The mRNA expression of HMGB1 **(C)** and TNF-α **(D)** were detected by real-time PCR. Glyceraldehyde-3-phosphate dehydrogenase (GAPDH) was used as the internal control gene. Data are representative of at least three independent experiments **(A–D)**.

1,25-Dihydroxyvitamin D plays a key role in sepsis (22, 25), and HMGB1 is a late mediator of endotoxin lethality released from macrophages, so we examined the effects of 1,25(OH)_2_D_3_ on HMGB1 secretion in macrophages. Data showed that LPS-induced HMGB1 secretion was suppressed by 1,25(OH)_2_D_3_ time course (Figure 1A) and various concentrations (Figure 1B) stimuli in bone marrow-derived macrophages (BMDMs). HMGB1 release was markedly attenuated by 1,25(OH)_2_D_3_ in a dose-dependent manner (Figure 1B). Here, we note that total HMGB1 expression is consistent, meaning that only the secretion of HMGB1 is suppressed by1,25(OH)_2_D_3_. 1,25(OH)_2_D_3_ also inhibited LPS-induced HMGB1 secretion in RAW264.7 cells, a murine macrophage cell line (Figure S1 in Supplementary Material). HMGB1 mRNA was further assayed by qPCR and showed no change in BMDMs between LPS stimulation alone and treatment of LPS plus 20 nM 1,25(OH)_2_D_3_ (Figure 1C). At the same time, TNF-α as a typical inflammatory factor was also detected and not affected by 1,25(OH)_2_D_3_ (Figure 1D). These observations suggest that 1,25(OH)_2_D_3_ modulates HMGB1 release independent on regulation of gene expression.

### 1,25(OH)_2_D_3_ Blocks LPS-Induced HMGB1 Nuclear Export in Macrophages

**Figure 2:**
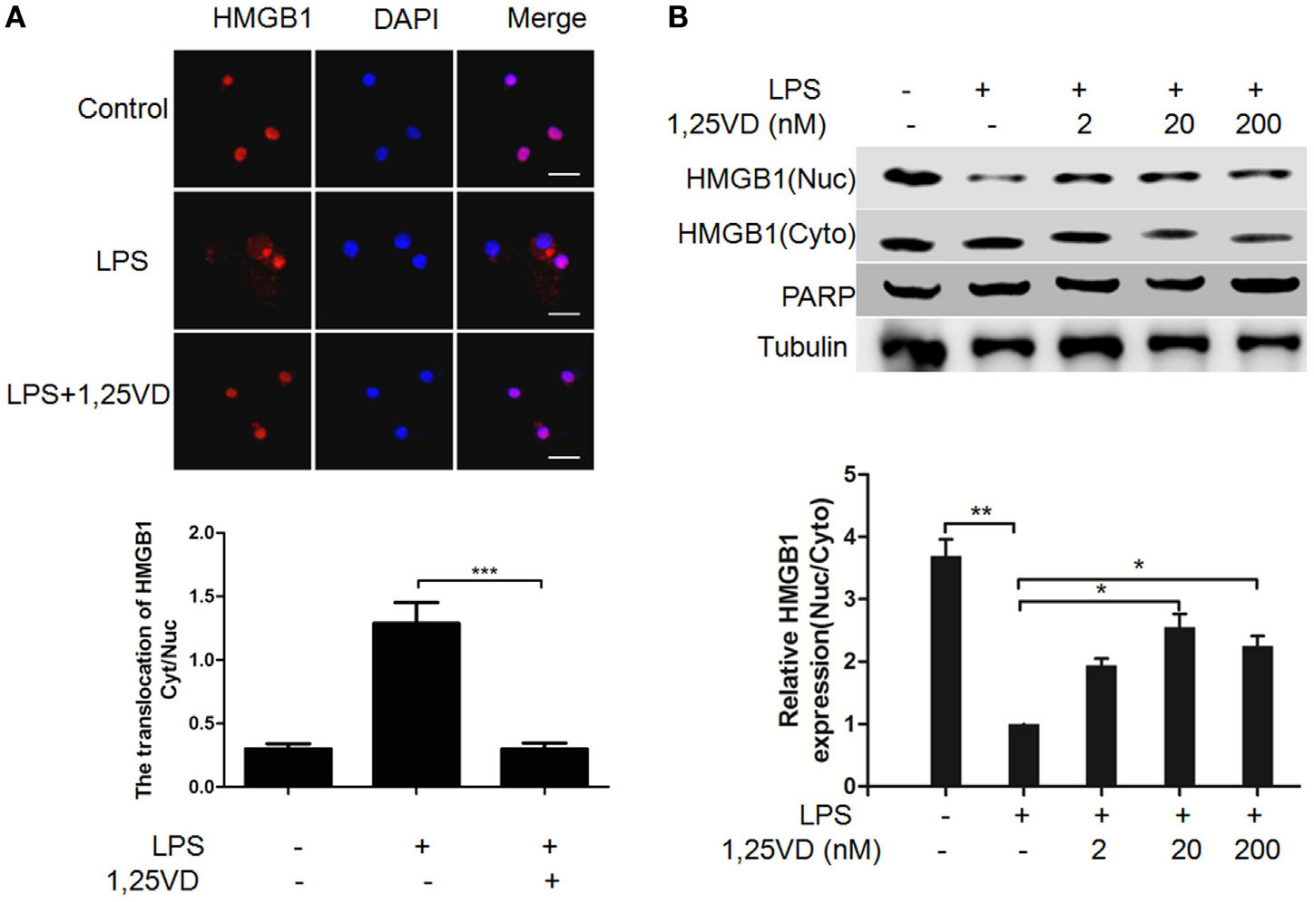
1,25-Dihydroxyvitamin D [1,25(OH)_2_D_3_] attenuates LPS-induced high-mobility group box 1 (HMGB1) nuclear export in macrophages. **(A)** LPS-induced HMGB1 nuclear export in present or absent with 1,25(OH)_2_D_3_ was detected by immunofluorescence. The cells were stained with anti-HMGB1 antibody (red), and the nuclei were visualized with 4′,6-diamidino-2-phenylindole (DAPI) staining (blue). Scale bar = 20 μm. More than 60 cells in each independent experiment were quantified. Data are representative of at least three independent experiments. Data are represented as mean ± SD. ***p < 0.001 versus LPS alone. **(B)** Bone marrow-derived macrophage cells were stimulated with various doses 1,25(OH)_2_D_3_ various doses plus LPS (100 ng/ml) as indicated. Nuclear (Nuc) and cytoplasmic (Cyto) proteins were isolated and presented by Western blotting with antibodies as indicated. PARP and tubulin were internal controls for the nuclear and cytoplasmic. Band intensity was quantified and calculated as indicated. Each bar is the mean of three independent experiments. Data are represented as mean ± SD. *p < 0.05; **p < 0.01; ***p < 0.001 versus LPS alone.

The translocation of HMGB1 from the nucleus to the cytoplasm can be induced by LPS, which is crucial for its release (6). To address the mechanism of 1,25(OH)_2_D_3_ regulation on HMGB1 secretion, we analyzed LPS-induced HMGB1 translocation. LPS-induced HMGB1 translocation from the nucleus to the cytoplasm was observed in Figure 2A, and its nuclear export was blocked with 1,25(OH)_2_D_3_ stimuli. Then, the distribution of HMGB1 was reconfirmed by subcellular fractionation. As shown in Figure 2B, a shift of HMGB1 from the nucleus to the cytoplasm was observed in BMDMs induced by LPS. 1,25(OH)_2_D_3_ promoted the amount of HMGB1 in nuclear in a dose-dependent manner, indicating 1,25(OH)_2_D_3_ blocks the HMGB1 nuclear export in macrophages.

### HO-1 Is Required for the Inhibition of 1,25(OH)_2_D_3_ on LPS-Induced HMGB1 Secretion

**Figure 3:**
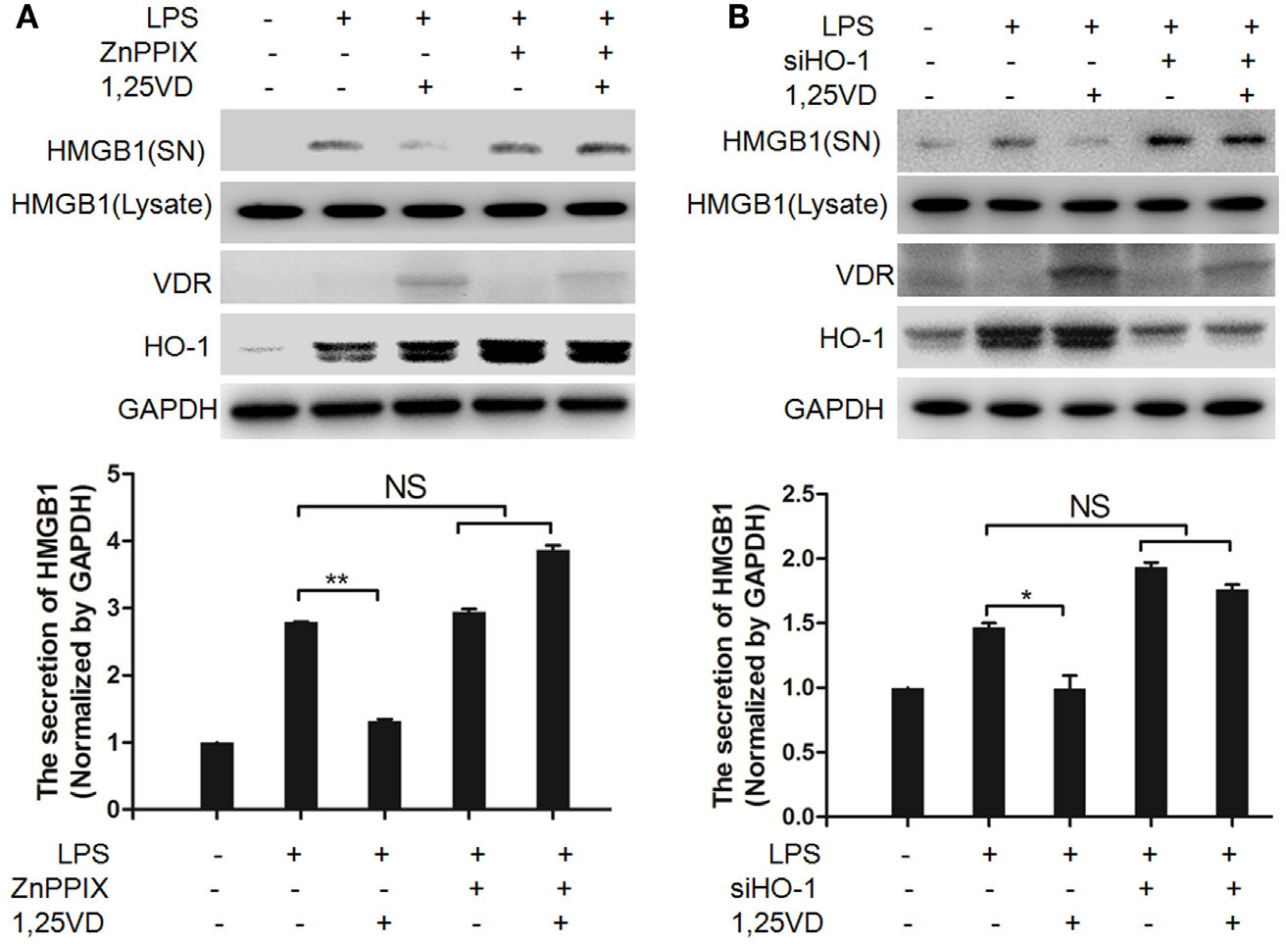
Hemeoxygenase-1 (HO-1) is required for 1,25-dihydroxyvitamin D [1,25(OH)_2_D_3_] inhibiting LPS-induced high-mobility group box 1 (HMGB1) secretion. **(A,B)** LPS pretreatment for 4 h followed by 1,25(OH)_2_D_3_ for 12 h with or without 5 nM ZnPPIX (HO-1 activity inhibitor) **(A)** or 100 nM siHO-1 **(B)**. Supernatants (SN) and cell extracts (Lysate) were analyzed by Western blotting. Band intensities were quantified from three independent experiments. Data are represented as mean ± SD. *p < 0.05, **p < 0.01 versus LPS alone.

Hemeoxygenase-1 has been reported to control HMGB1 nuclear translocation and block HMGB1 secretion (12). The HO-1 inhibitor ZnPPIX was used to examine whether HO-1 is involved in the blocking of HMGB1 secretion by 1,25(OH)_2_D_3_. We found that ZnPPIX indeed rescued the inhibition of HMGB1 secretion by 1,25(OH)_2_D_3_. Consistent with this result, the suppression of 1,25(OH)_2_D_3_ on LPS-induced HMGB1 secretion was also recovered by siRNA HO-1 (Figure 3B). Together, the above data indicated that HO-1 is important for the HMGB1 secretion inhibited by1,25(OH)_2_D_3_.

### 1,25(OH)_2_D_3_ Upregulates LPS-Induced HO-1 Expression in Macrophages

**Figure 4:**
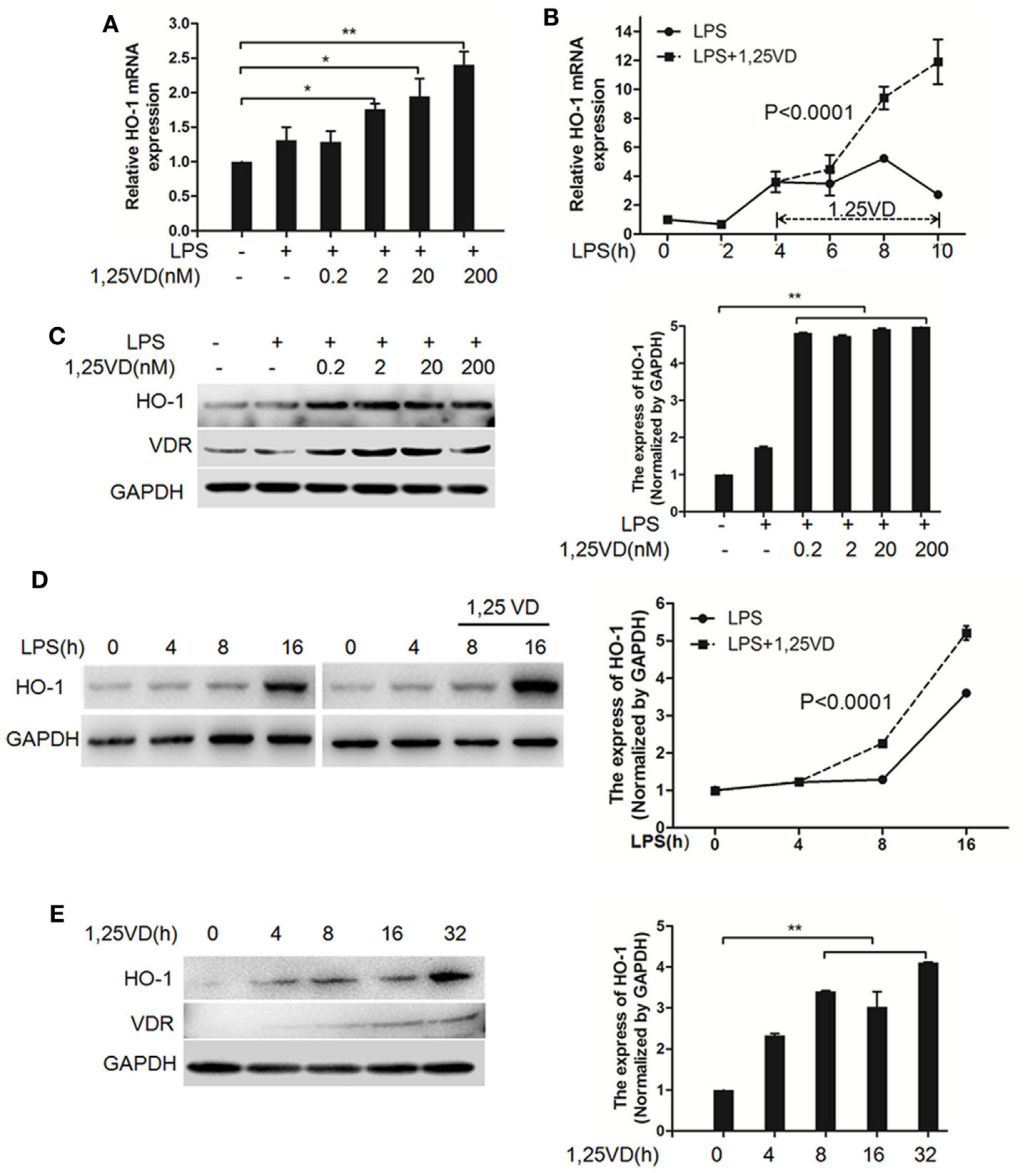
1,25-Dihydroxyvitamin D [1,25(OH)_2_D_3_] increases LPS-induced hemeoxygenase-1 (HO-1) expression in macrophages. **(A,B)** LPS-induced HO-1 mRNA expressions in bone marrow-derived macrophages (BMDMs) with 1,25(OH)_2_D_3_ **(A)** various dose treatment (2, 20, and 200 nM) or **(B)** time course (0, 2, 4, 6, 8, and 10 h) were detected by QPCR normalized to glyceraldehyde-3-phosphate dehydrogenase (GAPDH). Data are representative of at least three independent experiments. *p < 0.05, **p < 0.01 versus control, *p* < 0.0001 versus LPS alone. **(C,D)** The LPS-induced HO-1 protein expression in BMDMs in absent or present of 1,25(OH)_2_D_3_ **(C)** various dose (0.2, 2, 20, and 200 nM) or **(D)** time course (0, 4, 8, and 16 h) treatment. **(E)** The protein expression of HO-1 with 1,25(OH)_2_D_3_ time course (0, 4, 8, 16, and 32 h) treatment in BMDMs. Band intensities were quantified from three independent experiments **(C–E)**. Data are represented as mean ± SD. **p < 0.01 versus control, *p* < 0.0001 versus LPS alone.

Based on the above data, we next assessed whether 1,25(OH)_2_D_3_ regulates the induction of HO-1 induced by LPS. In BMDMs, HO-1 mRNA induction was observed after LPS stimulation, but there was much more induction with 1,25(OH)_2_D_3_ stress together (Figure 4A). Time-course studies confirmed that the induction of HO-1 transcript by 1,25(OH)_2_D_3_ was time dependent in BMDMs cells (Figure 4B). Consistently, similar regulations were seen for the protein levels of HO-1 (Figures 4C,D). Moreover, HO-1 expression also can be directly induced by 1,25(OH)_2_D_3_ alone (Figure 4E).

### 1,25(OH)_2_D_3_ Enhances Nrf2 Activation to Promote HO-1 Transcription

**Figure 5:**
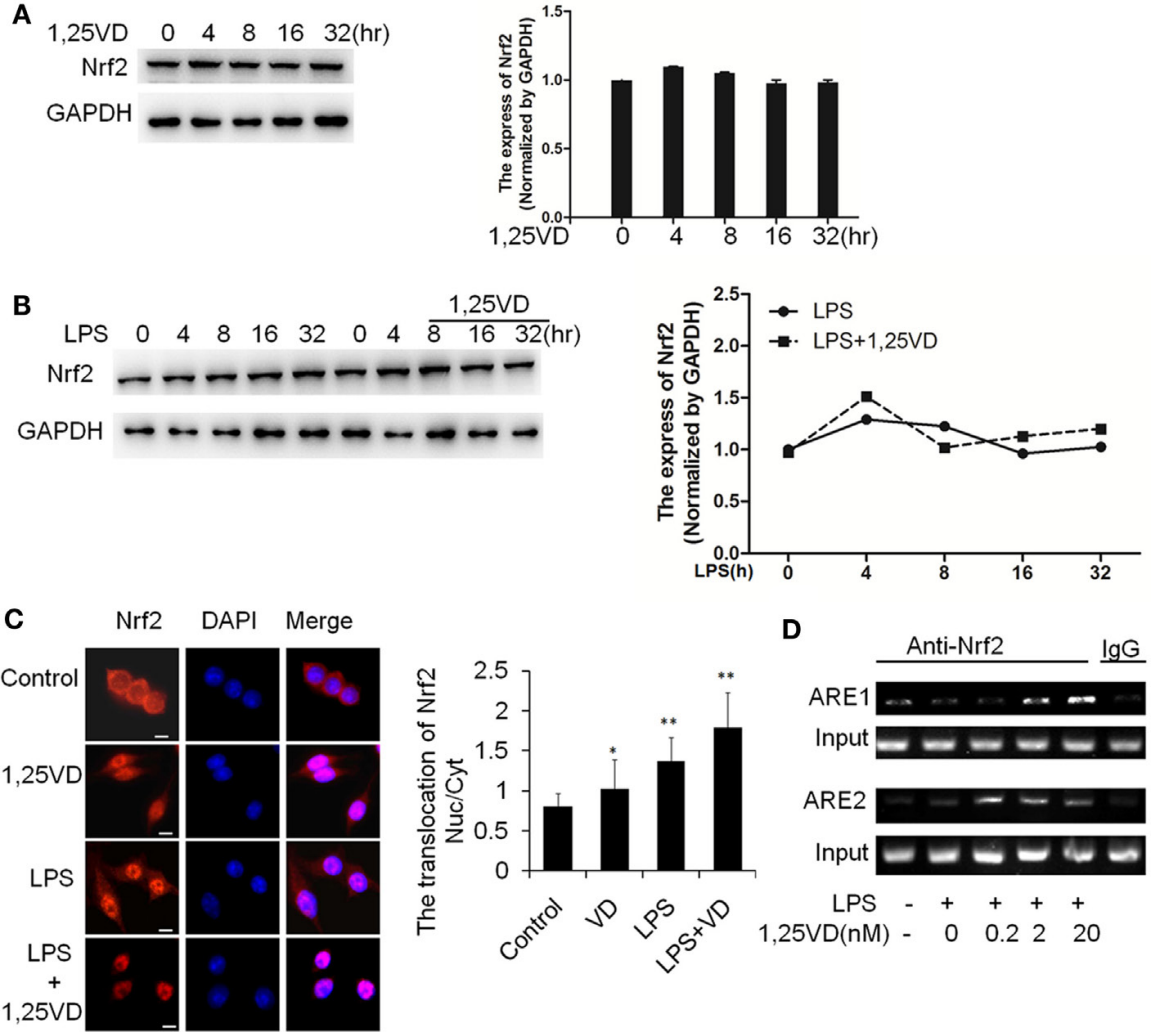
1,25-Dihydroxyvitamin D [1,25(OH)_2_D_3_] promotes hemeoxygenase-1 (HO-1) transcription *via* increasing NF-E2-related factor 2 (Nrf2) activation in macrophages. **(A)** The inductions of Nrf2 by the 1,25(OH)_2_D_3_ time course (0, 4, 8, 16, and 32 h) treatment were detected by Western blotting. **(B)** The induction of Nrf2 in LPS time course (0, 4, 8, 16, and 32 h) treatment in absent or present with 1,25(OH)_2_D_3_. Band intensities were quantified from three independent experiments. **(C)** Nrf2 translocation in absent or present with 1,25(OH)_2_D_3_ following LPS stimulation was detected by immunofluorescence. The cells were stained with anti-Nrf2 antibody (red), and the nuclei were visualized with 4′,6-diamidino-2-phenylindole (DAPI) staining (blue). Scale bar = 10 μm. More than 60 cells in each independent experiment were quantified. Each bar is the mean of three independent experiments. Data are represented as mean ± SD. *p < 0.05, **p < 0.01, ***p < 0.001 versus control. **(D)** Illustration of the ARE1 and ARE2 cis-DNA elements and the chromatin immunoprecipitation (ChIP) primers within the promoter of the HO-1 gene for ChIP assays. LPS promotes Nrf2 binding to the ARE site, and 1,25(OH)_2_D_3_ various dose treatment (0.2, 2, and 20 nM) enhances Nrf2 binding in bone marrow-derived macrophages. The DNA fragment precipitated by anti-Nrf2 antibody was assessed by regular-PCR. IgG as negative control. Data are representative of at least three independent experiments.

Because HO-1 is a proved target of Nrf2, we expected that 1,25(OH)_2_D_3_ might have an effect on Nrf2 expression. Indeed, there is no induction of Nrf2 on stimulation with LPS or LPS plus 1,25(OH)_2_D_3_ together in BMDMs (Figures 5A,B). Considering the main form of Nrf2 activation is nuclear translocation, we examined the subcellular localization of Nrf2 by immunofluorescent assay. 1,25(OH)_2_D_3_ or LPS could induce the Nrf2 nuclear translocation individually, but co-stimulation with 1,25(OH)_2_D_3_ markedly enhanced the LPS-induced Nrf2 nuclear translocation (Figure 5C). ARE1 and ARE2 are the Nrf2 cis-DNA elements identified in the mouse HO-1 gene promoter (26). Chromatin immunoprecipitation (ChIP) assays showed that Nrf2 binding to these sites was increased by the 1,25(OH)_2_D_3_ stimuli in dose-dependent manner (Figure 5D). Together, these data demonstrate that 1,25(OH)_2_D_3_ increases Nrf2 nuclear translocation and promotes the transcript of HO-1 expression.

### 1,25(OH)_2_D_3_ Analog Alleviates LPS-Induced Sepsis

**Figure 6:**
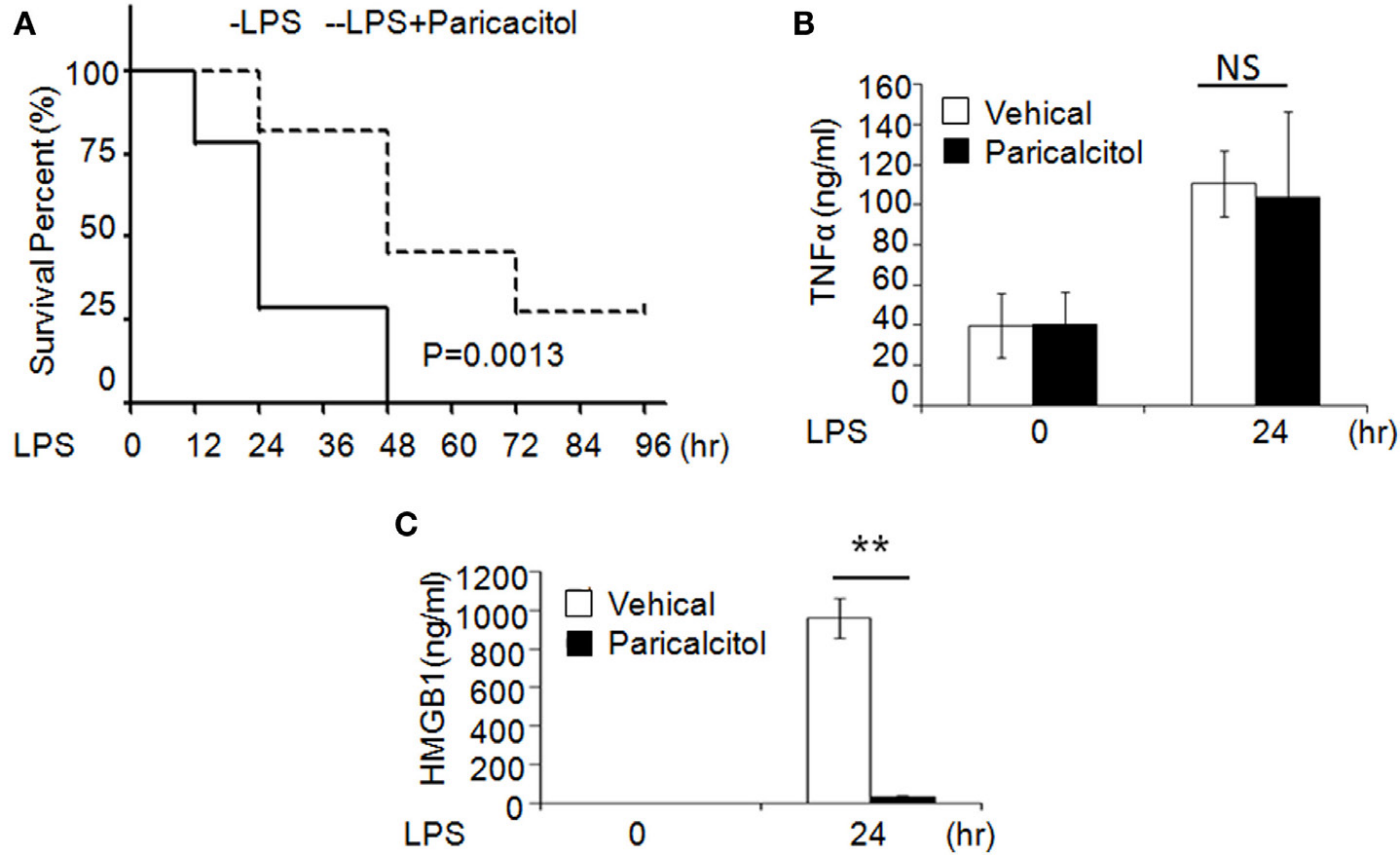
1,25-Dihydroxyvitamin D analog treats LPS-induced sepsis in mice. **(A)** Survival curves of mice with the vehicle and paricalcitol treatment after i.p. injection of LPS at 20 mg/kg; *n* = 7–8. *p* = 0.0013 by log-rank test. **(B,C)** Serum TNF-α **(B)** and high-mobility group box 1 (HMGB1) **(C)** concentration in mice at 0 and 24 h after LPS challenge were analyzed by ELISA. **p < 0.01, *p* = 0.0013 versus vehicle.

To validate the role of 1,25(OH)_2_D_3_ in sepsis, we induced murine sepsis by LPS and treated either with vehicle or 1,25(OH)_2_D_3_ as described below. Four hours after the LPS (20 mg/kg i.p.) injection, mice were treated with the vehicle (control group) or the non-calcemic vitamin D analog paricalcitol [1,25(OH)_2_D_3_ not used for its side-effect of hypercalcemia]. After LPS injection, 75% of control group mice died within 24 h and all died by 48 h; by contrast, 75% of the paricalcitol-treated mice survived within 24 h and 25% of them still survived after 96 h (Figure 6A). Consistent with our finding, within 24 h of LPS treatment, the serum HMGB1 was significantly decreased in the drug group (Figure 6B), but the serum TNF-α had no much change compared the drug group with control group (Figure 6C). These data indicate that the survival rate of LPS-induced sepsis can be improved by 1,25(OH)_2_D_3_ *via* blocking HMGB1 secretion.

## Discussion

**Figure 7:**
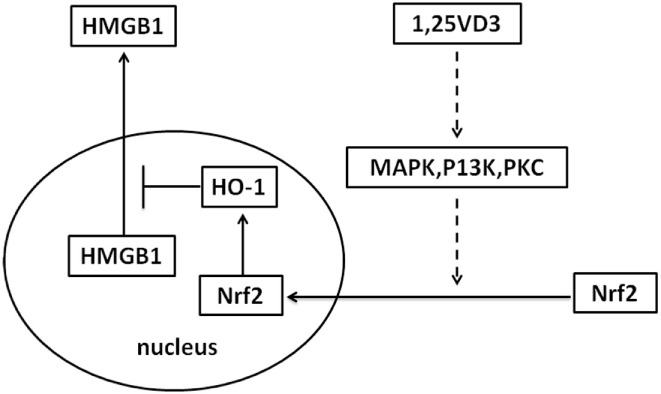
Proposed mechanism whereby vitamin D inhibits high-mobility group box 1 (HMGB1) secretion *via* blocking the hemeoxygenase-1 (HO-1)/NF-E2-related factor 2 (Nrf2) pathway.

Sepsis is a common indication for ICU admission and is associated with marked morbidity and mortality. A number of observational studies have shown low vitamin D levels related to the risk of sepsis (27–29). However, the effectiveness of vitamin D supplementation in sepsis treatment is contradictory in clinical trial (29, 30). In our study, the treatment with paricalcitol in mouse model of LPS-induced sepsis increased the survival rate of mice and decreased the LPS-induced HMGB1 secretion in serum (Figure 6). As a result, our study provides novel information on the role of vitamin D in sepsis *via* the Nrf2–HO-1–HMGB1 pathway (Figure 7).

Pretreatment with the non-calcemic vitamin D analog paricalcitol was previously reported by us to suppress LPS-induced inflammation *via* the MicroRNA-155–SOCS1 pathway (31), which suggested that vitamin D has a good preventive effect on sepsis. But the therapeutic effect of 1,25(OH)_2_D_3_ for sepsis is not clear till now. In this study, we found that the concentration of plasma HMGB1 was significantly decreased with paricalcitol treatment. HMGB1 secretion from macrophages mediates potent anti-inflammatory effects (2, 3, 9). Consistently, vitamin D supplementation for 12 weeks reduced the HMGB1 concentration in elderly women reported by Gmiat et al. (32). Leaf et al. tried vitamin D supplementation in sepsis in clinical trial, and calcitriol administration had no effects on immunomodulatory factors TNF-α or IL-6, but can increase IL-10 expression (33). Our finding also verified that vitamin D has no effect on TNF-α expression, but HMGB1 as a new regulation factor by 1,25(OH)_2_D_3_ came to light.

The inhibition of HMGB1 secretion by 1,25(OH)_2_D_3_ was due to block the nuclear export of HMGB1 (Figure 2). HMGB1 cytoplasmic translocation is controlled by HO-1 (12). Here, we showed that the level of HO-1 expression was upregulated by 1,25(OH)_2_D_3_ (Figure 4). Consistent with our finding, 1,25(OH)_2_D_3_ was reported to upregulate HO-1 expression in glial cells (34), and HO-1 expression was decreased in vitamin D deficiency in obese rats (35). Then, the important role of HO-1 in inhibition of HMGB1 secretion by 1,25(OH)_2_D_3_ was testified by interference experiment with HO-1 inhibitor or siHO-1 (Figure 3). Nrf2 is a crucial transcription factor for HO-1. We further found that Nrf2 can be activated by 1,25(OH)_2_D_3_ and bound to the promoter of HO-1 (Figure 5) in macrophage. The Nrf2/ARE pathway can be activated by posttranscriptional activating Nrf2 *via* phosphorylation by signaling protein kinases (PKC, MAPKs, and/or PI-3-K) (36–39). Meanwhile, 1,25(OH)_2_D_3_ can regulate PKC, MAPKs, and/or PI-3-K pathways (40–42). These report generate clues for the mechanism of the regulation of 1,25(OH)_2_D_3_ on the Nrf2 activation. In short, the possible modes of vitamin D inhibiting LPS-Induced HMGB1 secretion *via* targeting the Nrf2–HO-1–HMGB1 pathway in Macrophages are represented in Figure 7. However, the precise regulation of 1,25(OH)_2_D_3_ in the Nrf2–HO-1–HMGB1 pathway remains unclear, and the detailed molecular mechanism needs to be fully defined in future studies.

## Materials and Methods

### Animals and Treatment

All mice were from a C57BL/6 background. This study was carried out in accordance with the recommendations of the guidelines of the Animal Care Committee of Nanjing Medical University. The protocol was approved by the Animal Care Committee of Nanjing Medical University. Mice were used experimentally at 2–4 months of age. To induce sepsis, we injected mice with one dose of LPS (O111:B4, Sigma L2630; 10 mg/kg i.p.). We treated mice with vehicle (60:30:10 propylene glycol:water:ethanol) or the non-calcemic vitamin D analog paricalcitol (19-nor-1,25-dihydroxyvitamin D2, 200 ng/kg; provided by Abbott Laboratories) after an LPS (20 mg/kg) challenge. Blood was collected for serum cytokine measurement from the tail vein at the indicated times after LPS treatment.

### Reagents and Cytokine Quantization

ZnPPIX was bought from Sigma. TNF-α and HMGB1 concentrations in the serum or culture media were determined by ELISA using commercial ELISA kits obtained from Bio-Legend (San Diego, CA, USA). Data were analyzed by Student’s *t*-test. A *p*-value <0.05 was considered statistically significant.

### Cell Culture and Treatment

L929 and RAW264.7 cells were grown in DMEM supplemented with 10% FBS. BMDMs were cultured as described previously (43). In brief, mouse bone marrow cells were plated in DMEM supplemented with 10% FBS. After overnight culture, the unattached cells were re-plated and differentiated into BMDMs in 30% L929 conditioned media. Cells cultures were usually treated with 100–200 ng/ml LPS with or without 1,25(OH)_2_D_3_ treatment as specified in each experiment, followed by the isolation of total RNAs, lysates, or media supernatants (SN) for various assays.

### Western Blot Analysis

To analyze the secretion of HMGB1 in the SN, culture media were replaced with 2% serum DMEM (Gibco by Life Technologies) concentrated with ethanol. After removing cell debris, Western blot analysis was performed. Briefly, equal amounts of protein were separated by 8–12% SDS-PAGE and electrotransferred onto polyvinylidene difluoride membranes. The Abs used in this study included HMGB1 (ab79823, rabbit monoclonal; Abcam); VDR (sc-13133, mouse monoclonal; Santa Cruz); glyceraldehyde-3-phosphate dehydrogenase (GAPDH) (NP-002037, mouse monoclonal; ZSGB-BIO); HO-1 (sc-10789, rabbit polyclonal; Santa Cruz); and Nrf2 (sc-722, rabbit polyclonal; Santa Cruz); F4/80(12-4801-82, mouse monoclonal, eBioscience).

### Immunostaining

Cells were incubated with 4% paraformaldehyde in PBS for 20 min and then washed with PBS and permeabilized with 0.02% NP-40/PBS for 10 min. The cells were washed and incubated with serum for 4 h and then incubated with anti-HMGB1 antibody overnight at 4°C. The cells were gently washed with PBS, followed by incubation with Cy3-labeled secondary antibody for 90 min at room temperature. The cells were mounted with an emulsion oil solution containing 4′,6-diamidino-2-phenylindole (DAPI) after washing with PBS.

### Reverse Transcription and Real-time PCR

Total RNA was extracted from cells using the TRIzol reagent (catalog no. 15596-018; Life Technologies). Reverse transcription reactions were performed with the PrimeScript™ RT Master Mix cDNA Synthesis Kit (catalog no. RR036A, Takara Bio). Real-time PCR was performed using SYBR Mix reagents (catalog no. A5303, Takara Bio). Amplification conditions were as follows: 95°C (5 min) followed by 40 cycles of 95°C (10 s), 60°C (30 s). The sequences of PCR primers used were listed in Table 1. GAPDH was used as the internal control gene.

**Table 1:** Primers in this study.

Primers	Sequences 5′-3′
Mouse TNF-a-1	TCAGCCTCTTCTCATTCCTG
Mouse TNF-a-2	CAGGCTTGTCACTCGAATTT
Mouse IL-6-1	ATAGTCCTTCCTACCCCAATTTCC
Mouse IL-6-2	CTGACCACAGTGAGGAATGTCCAC
m-Hmox1-1	AAGCCGAGAATGCTGAGTTCA
m-Hmox1-2	GCCGTGTAGATATGGTACAAGGA
mNrf2-1	TGGACGGGACTATTGAAGGCTG
mNrf2-2	GCCGCCTTTTCAGTAGATGGAGG
mhmgb1-1	GGCGAGCATCCTGGCTTATC
mhmgb1-2	GGCTGCTTGTCATCTGCTG
mgapdh-1	GGTCTACATGTTCCAGTATGACTCCAC
mgapdh-2	GGGTCTCGCTCCTGGAAGAT
HO-1 siRNA	UUACAUGGCAUAAAUUCCCACUGCC

### Preparation of Cytoplasm and Nuclear Extracts

Cytoplasm and nuclear extracts were prepared using the Nuclear and Cytoplasm Protein Extraction Kit according to the manufacturer’s instructions (Beyotime Institute of Biotechnology). Briefly, cells were scraped off, washed in ice-cold PBS, and then resuspended in 200 µl of ice-cold cytoplasm extraction buffer A with 1 mM PMSF, 1 mM Na_4_VO_3_, and protease inhibitor mixture. After incubation with cytoplasm extraction buffer B for 1 min in ice bath and following vortexing for 5 s, cell lysates were centrifuged at 12,000 × *g* for 5 min at 4°C. SN were aliquoted and stored at −80°C. Nuclear pellets were resuspended in 50 µl of nuclear extraction buffer. After 15 sets of vortexing for 15 s every 2 min at 4°C, lysates were centrifuged at 12,000 × *g* for 10 min at 4°C. Nuclear extracts were aliquoted and stored at −80°C until use.

### Chromatin Immunoprecipitation

Protein binding to the HO-1 regulatory genomic region was assessed by a ChIP assay, according to the manufacturer’s instructions (Upstate, Lake Placid, NY, USA). Briefly, BMDMs were homogenized, and DNA-associated proteins were dual cross-linked in 1% formaldehyde/2.5 mM EGS (ethylene glycol-bis, Sigma) in PBS with protease inhibitors (Sigma), as described in reference (44), Using anti-Nrf2 antibody. The ARE1 region of HO-1 was amplified with the primers 5′-TGAAGTTAAAGCCGTTCCGG and 3′-AGCGGCTGGAATGCTGAGT; the ARE2 region was amplified with the primers 5′-GGGCTAGCATGCGAAGTGAG and 3′-AGACTCCGCCCTAAGGGTTC.

### Statistical Analysis

Statistical comparisons were carried out using unpaired two-tailed Student’s *t*-test and one-way ANOVA or two-way ANOVA followed by Tukey’s *post hoc* test analysis of variance as appropriate, with *p* < 0.05 being considered statistically significant. For the time to mortality in mice with LPS, we estimated the survival curves according to paricalcitol group with the use of the Kaplan–Meier method and compared the results by means of the log-rank test. Data values were presented as the mean ± SEM.

## Ethics Statement

This study was carried out in accordance with the recommendations of the guidelines of the Animal Care Committee of Nanjing Medical University, Jiangsu, China. The protocol was approved by the Animal Care Committee of Nanjing Medical University.

## Author Contributions

YC and ZR designed the research, analyzed data, and wrote the paper; NZ, NX, YP, MX, and JW provided research reagents and technical assistance; SY and HZ assisted in data analysis and manuscript preparation; YC was responsible for the overall research design, data analysis, and paper preparation.

## Conflict of Interest Statement

The authors declare that the research was conducted in the absence of any commercial or financial relationships that could be construed as a potential conflict of interest.

## Supplementary Material

Figure S1 1,25-dihydroxyvitamin D [1,25(OH)_2_D_3_] blocks LPS-induced high-mobility group box 1 (HMGB1) secretion in RAW264.7 cells. RAW264.7 cells treated with various dose 1,25(OH)_2_D_3_ (0.2, 2, 20, and 200 nM) in present of LPS (100 ng/ml) in RAW264.7 cells.

Figure S2 Negative control for anti-NF-E2-related factor 2 (Nrf2) and anti-high-mobility group box 1 (HMGB1) in immunofluorescence with isotype antibody. The cells were stained with isotype antibody and second antibody (red), the nuclei were visualized with 4′,6-diamidino-2-phenylindole (DAPI) staining (blue). Scale bar = 20 μm.

Figure S3 No LPS contaminated in L929 medium. LPS was detected in L929 medium, DMEM (negative control) and DMEM with 200 ng/ml LPS (positive control) by LPS ELISA kit (Genscript).

Figure S4 LPS-induced high-mobility group box 1 (HMGB1) nuclear translocation is blocked by 1,25-dihydroxyvitamin D [1,25(OH)_2_D_3_] in bone marrow-derived macrophages maintained with 20 ng/ml MCSF (PeproTech). LPS-induced HMGB1 translocation in absent or present of 1,25(OH)_2_D_3_ was detected by immunofluorescence. The cells were stained with anti-HMGB1 antibody (red), and the nuclei were visualized with 4′,6-diamidino-2-phenylindole (DAPI) staining (blue). Scale bar = 20 μm.

Figure S5 Phenotypic analyses of the tissues (lung, liver, and kidney) of mouse with the vehicle and paricalcitol treatment and i.p. injection of LPS for 24 h. **(A)** Representative H&E staining in lung, liver, and kidney. Immunostaining with anti-F4/80 (green) and anti-high-mobility group box 1 (HMGB1) (red), anti-F4/80 (green) and anti-HO-1 (red), anti-F4/80 (green) and anti-NF-E2-related factor 2 (Nrf2) in lung **(B)**, liver **(C)**, and kidney **(D)**. Scale bar = 100 µm.

## Abbreviations

1,25(OH)_2_D_3_, 1,25-dihydroxyvitamin D; BMDMs, bone marrow-derived macrophages; ChIP, chromatin immunoprecipitation; HMGB1, high-mobility group box 1; HO-1, hemeoxygenase-1; Nrf2, NF-E2-related factor 2.
