# Corrigendum: 1,25-Dihydroxyvitamin D Inhibits LPS-Induced High-Mobility Group Box 1 (HMGB1) Secretion *via* Targeting the NF-E2-Related Factor 2–Hemeoxygenase-1–HMGB1 Pathway in Macrophages

**DOI:** 10.3389/fimmu.2018.00357

**Published:** 2018-03-07

**Authors:** Zebing Rao, Na Zhang, Ning Xu, Ying Pan, Mengjun Xiao, Junxian Wu, Hong Zhou, Shuo Yang, Yunzi Chen

**Affiliations:** ^1^Department of Immunology, Nanjing Medical University, Nanjing, China; ^2^Key Laboratory of Antibody Techniques of Ministry of Health, Nanjing Medical University, Nanjing, China; ^3^Department of Pathology, Nanjing Medical University, Nanjing, China; ^4^Medical Centre for Digestive Diseases, Second Affiliated Hospital of Nanjing Medical University, Nanjing, China

**Keywords:** sepsis, vitamin D, inflammation, high-mobility group box 1 protein, damage-associated molecular patterns

In the original article “1,25-Dihydroxyvitamin D Inhibits LPS-Induced HMGB1 Secretion *via* Targeting the Nrf2-HO-1-HMGB1 Pathway in Macrophages”, we neglected to include the funder, National Natural Science Foundation of China grant (81570499) to Shuo Yang. The authors apologize for this error and state that this does not change the scientific conclusions of the article in any way.

The original article has been updated.

## Conflict of Interest Statement

The authors declare that the research was conducted in the absence of any commercial or financial relationships that could be construed as a potential conflict of interest.

