# Supplementary figures and images for: 1,25-Dihydroxyvitamin D Inhibits LPS-Induced High-Mobility Group Box 1 (HMGB1) Secretion via Targeting the NF-E2-Related Factor 2–Hemeoxygenase-1–HMGB1 Pathway in Macrophages
Source: Front Immunol. 2017 Oct 18;8:1308. doi: 10.3389/fimmu.2017.01308 (PMC5650703; doi:10.3389/fimmu.2017.01308)

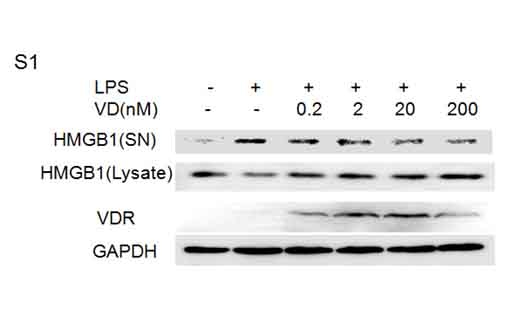

Supplement: Figure S1 — 1,25-dihydroxyvitamin D [1,25(OH)2D3] blocks LPS-induced high-mobility group box 1 (HMGB1) secretion in RAW264.7 cells. RAW264.7 cells treated with various dose 1,25(OH)2D3 (0.2, 2, 20, and 200 nM) in present of LPS (100 ng/ml) in RAW264.7 cells. [file Image_1.JPEG]

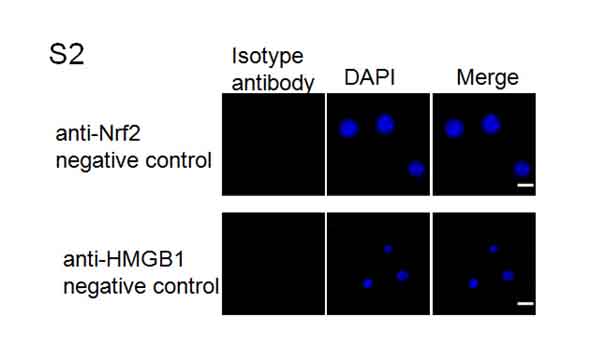

Supplement: Figure S2 — Negative control for anti-NF-E2-related factor 2 (Nrf2) and anti-high-mobility group box 1 (HMGB1) in immunofluorescence with isotype antibody. The cells were stained with isotype antibody and second antibody (red), the nuclei were visualized with 4′,6-diamidino-2-phenylindole (DAPI) staining (blue). Scale bar = 20 μm. [file Image_2.JPEG]

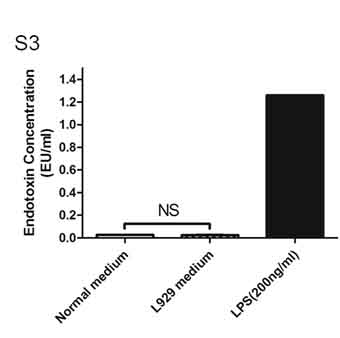

Supplement: Figure S3 — No LPS contaminated in L929 medium. LPS was detected in L929 medium, DMEM (negative control) and DMEM with 200 ng/ml LPS (positive control) by LPS ELISA kit (Genscript). [file Image_3.JPEG]

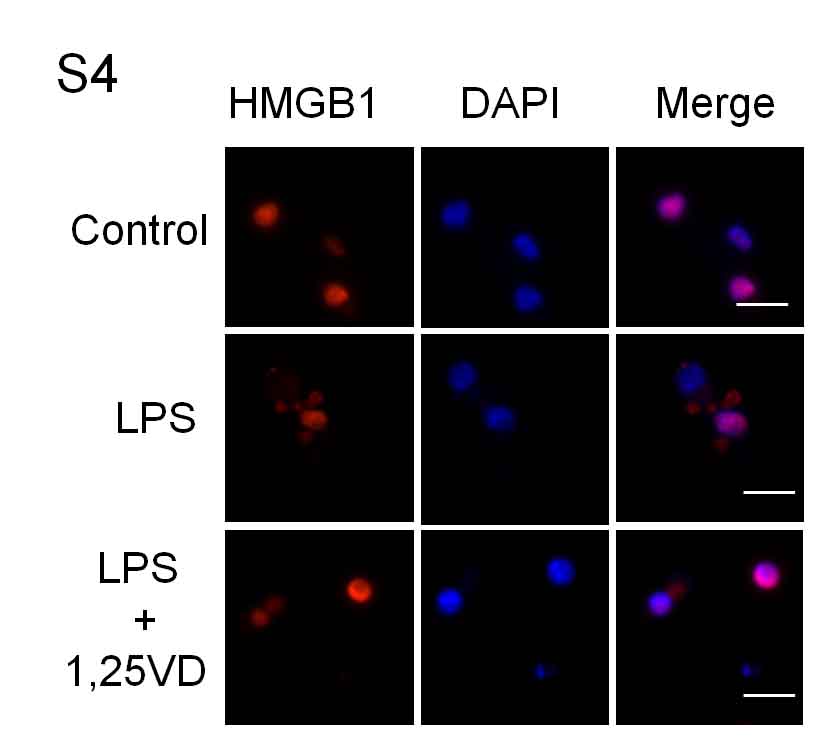

Supplement: Figure S4 — LPS-induced high-mobility group box 1 (HMGB1) nuclear translocation is blocked by 1,25-dihydroxyvitamin D [1,25(OH)2D3] in bone marrow-derived macrophages maintained with 20 ng/ml MCSF (PeproTech). LPS-induced HMGB1 translocation in absent or present of 1,25(OH)2D3 was detected by immunofluorescence. The cells were stained with anti-HMGB1 antibody (red), and the nuclei were visualized with 4′,6-diamidino-2-phenylindole (DAPI) staining (blue). Scale bar = 20 μm. [file Image_4.JPEG]

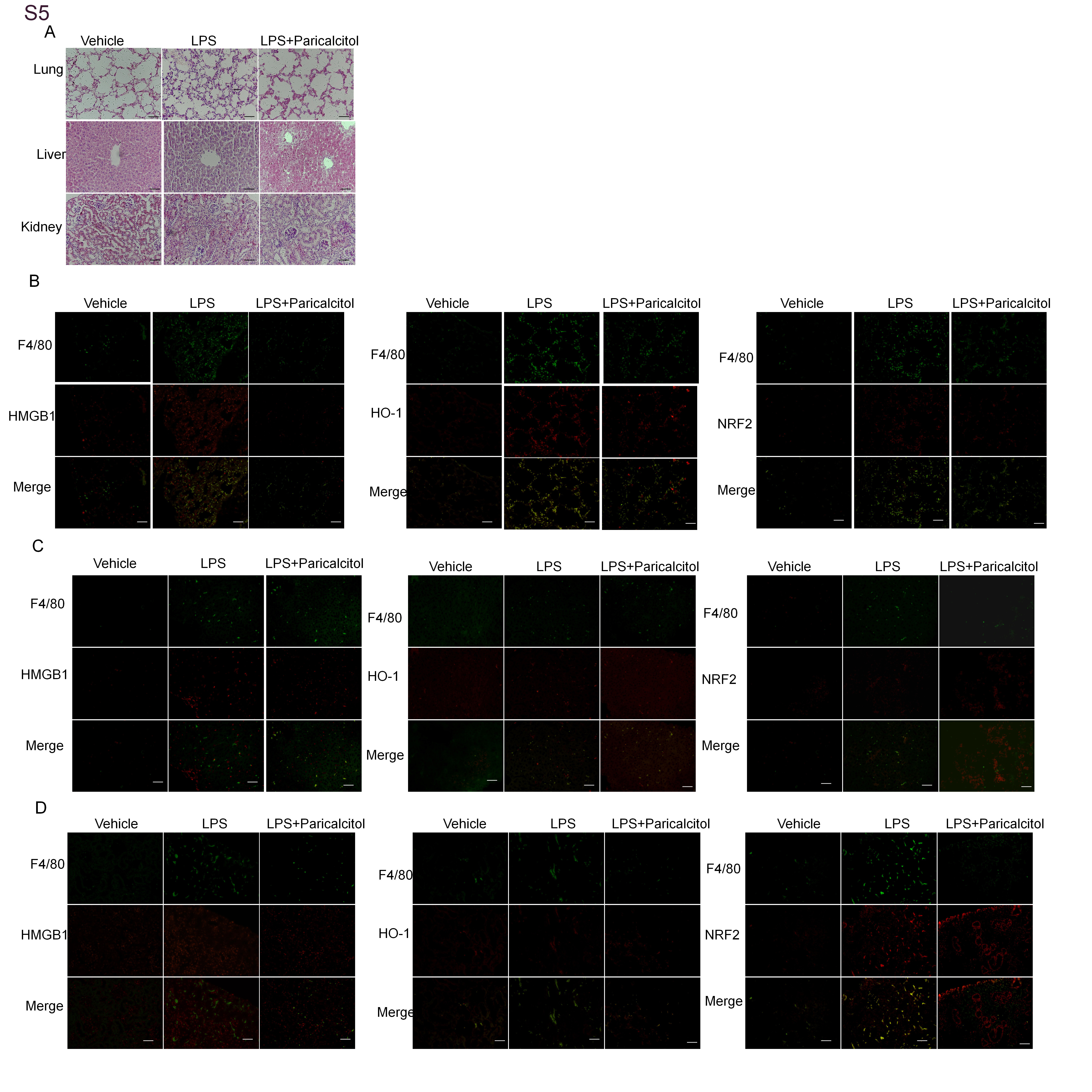

Supplement: Figure S5 — Phenotypic analyses of the tissues (lung, liver, and kidney) of mouse with the vehicle and paricalcitol treatment and i.p. injection of LPS for 24 h. (A) Representative H&E staining in lung, liver, and kidney. Immunostaining with anti-F4/80 (green) and anti-high-mobility group box 1 (HMGB1) (red), anti-F4/80 (green) and anti-HO-1 (red), anti-F4/80 (green) and anti-NF-E2-related factor 2 (Nrf2) in lung (B), liver (C), and kidney (D). Scale bar = 100 µm. [file Image_5.JPEG]
